# Informal Caregivers' Role in Care of Assisted Living Residents with Advanced Dementia and Its impact on These Providers' Mental Well‐Being

**DOI:** 10.1002/alz70861_108972

**Published:** 2025-12-23

**Authors:** Molly Perkins, Candace Lynn Kemp, Michael Lepore, Kenneth Hepburn, Carolyn K Clevenger, Mi‐Kyung M Song, Miranda A Moore, Alexis A Bender, Regine Haardoerfer, Regina A Shih

**Affiliations:** ^1^ Emory University, Atlanta, GA USA; ^2^ Emory Roybal Center for Dementia Caregiving Mastery, Atlanta, GA USA; ^3^ Birmingham/Atlanta VA GRECC, Atlanta, GA USA; ^4^ Georgia State University, Atlanta, GA USA; ^5^ University of Massachusetts Amherst, Amherst, MA USA

## Abstract

**Background:**

Informal caregivers (relatives or friends) of persons with dementia experience higher levels of stress and depressive symptoms compared with non‐dementia caregivers. Many of these heavily burdened caregivers opt for assisted living (AL) care to ease this burden. In most states in the U.S., AL, a social model of care, is not licensed as a medical facility and provides no 24‐hour skilled nursing care. Informal caregivers play critical roles in supplementing care in AL, yet we know little about how this supplemental care impacts informal caregiver’s well‐being. This study reports preliminary baseline findings from a 5‐year NIA‐funded (RF1AG069114/R01AG069114) longitudinal project designed to address this knowledge gap. In this analysis, we sought to characterize informal caregiver burden, involvement and mental well‐being.

**Method:**

We conducted baseline surveys, including qualitative open‐ended questions, with 134 informal caregivers of residents with advanced dementia in 39 AL settings in Georgia. Measures included the Zarit Burden Interview (ZB‐12), physical and mental well‐being (SF12), Frequency of care involvement (6‐items), and the Dementia Knowledge Assessment Tool (DKAT2). We calculated descriptive statistics to characterize the sample and conducted qualitative thematic analysis.

**Result:**

Most participants (mean age = 61) were female (85%), Non‐Hispanic White (84%), and had some college education or higher (91%). Close to half (45%) reported mild to moderate burden and more than one‐third (36%) reported severe burden. On average, mental well‐being (mean = 31.07) was below U.S. population norms. Frequency of care involvement in a typical week showed a relatively high level of involvement (mean = 19.55, range 0‐30, with higher scores indicating higher levels of involvement). Despite high levels of education, the average percentage of correct answers on the DKAT2 was below 75% (mean = 71.69), which may contribute to poor mental well‐being. Qualitative data showed that additional factors that may negatively impact caregivers’ mental well‐being include limited time for self‐care, staff turnover and lack of continuity of care, and poor or inconsistent communication with AL staff and hospice.

**Conclusion:**

These findings elucidate some of the unique challenges informal caregiver’s experience in the AL setting and point to possible areas for intervention to improved informal caregiver well‐being.

